# Five‐year clinical study of a novel porcine xenograft for anterior cruciate ligament reconstruction: Positive safety and performance outcomes

**DOI:** 10.1002/jeo2.70433

**Published:** 2025-09-23

**Authors:** Neil Hunt, Gabriel Oliver, Alberto Francés Borrego, William S. Pietrzak

**Affiliations:** ^1^ Fortius Clinic London UK; ^2^ Hospital Universitari de Bellvitge Barcelona Spain; ^3^ Hospital Clínico San Carlos Madrid Spain; ^4^ Department of Biomedical Engineering University of Illinois Chicago Illinois USA

**Keywords:** anterior cruciate ligament, immunological, porcine, reconstruction, xenograft

## Abstract

**Purpose:**

Many graft choices exist for anterior cruciate ligament (ACL) reconstruction including autograft, allograft and, to a lesser extent, synthetic graft, with all having significant limitations. While xenograft can circumvent many of these limitations, potential immunogenic response remains a concern. A novel decellularization process has been developed to remove the principal immunogenic epitopes from porcine digital extensor tendon to produce a nonimmunogenic, biomechanically appropriate ACL xenograft for clinical use. This study reports the first in‐human series utilising this xenograft.

**Methods:**

This was a 5‐year study of 40 patients, mean age 31.9 years (range: 18–48), 70% male, with mean follow‐up of 41.9 months (6–60 months) and 19 reaching 5 years. Radiographic and MRI analysis was performed as were a variety of clinical assessments, including arthrometric measurement of anterior tibial translation, Lachman test, Pivot Shift test, International Knee Documentation Committee (IKDC) Subjective questionnaire, and Knee Injury and Osteoarthritis Outcome Score (KOOS) questionnaire. All adverse events were recorded. Clinical outcomes were compared to those reported in the literature for autograft and/or allograft.

**Results:**

Adverse events included six graft ruptures which were limited to young males. No evidence of an immunogenic response was noted. Clinical outcome assessments and imaging analysis were in line with those reported in the literature for autograft and/or allograft.

**Conclusions:**

This first in‐human study of a novel porcine ACL xenograft demonstrated that it is biomechanically and immunologically suitable for clinical use with no safety concerns evident.

**Level of Evidence:**

Level IV.

## Graphical abstract

Porcine digital extensor tendon (p‐DET) xenograft was used for anterior cruciate ligament (ACL) reconstruction in 40 patients with follow‐up to 5 years. There was significant improvement in a variety of outcome measures after 3‐6 months which were maintained to 5 years. Six (15%) retears occurred in young males. The overall results suggest that this xenograft is a viable option for ACL reconstruction.

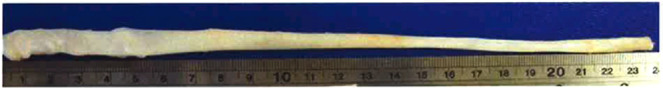

**Abbreviations**
ACL: anterior cruciate ligament
ANOVA: analysis of variance
BMI: body mass index
BPTB: bone‐patellar‐tendon bone
d‐pDET: decellularized porcine digital extensor tendon
IKDC: International Knee Documentation Committee
KOOS: Knee Osteoarthritis and Injury Outcome Score
MCL: medial collateral ligament
pDET: porcine digital extensor tendon
QoL: quality of life
SSD: side to side difference

## INTRODUCTION

Anterior cruciate ligament (ACL) tears account for 50%, or more, of all knee injuries with delays in treatment leading to increased risk of meniscal tears and early knee osteoarthritis [30, 37, 46]. ACL reconstruction is considered the best treatment option [33]. It is estimated that 400,000 ACL procedures are performed in the United States and up to 30,000 in the UK annually [5, 33].

There are several classes of ACL graft types available with none offering ideal characteristics. Autografts possess the best biological properties, but donor site morbidity is a major limitation [27]. Allografts obviate intraoperative graft harvesting but have higher failure rates and incorporate more slowly than autograft with the most desirable being from young, healthy donors that can be in short supply [27]. Synthetic grafts can be engineered to be reproducible but clinical success has been limited due, in part, to inferior mechanical properties and production of wear debris and biological reaction [28]. Xenografts can circumvent many of these issues, but one obstacle is the potential for discordant immunogenic response leading to rejection [14, 50]. However, an ACL xenograft processed to significantly reduce, or eliminate, immunogenicity would be an attractive alternative [20].

Cells of almost all mammals, except for humans and Old‐World primates, contain α‐Gal epitope (Galα1‐3Galβ1‐4GlcNAc‐R) which interacts with human anti‐Gal antibody and is responsible for approximately 95% of the associated xenograft immune response with non‐α‐Gal factors responsible for the remainder [15, 51]. Early clinical studies of bovine xenograft for ACL reconstruction showed high rates of synovitis, rupture, and infection [8, 17, 55]. More recently, attention has been directed toward porcine ACL xenografts utilising improved processing methodology to ameliorate the immunogenic response. Methods to remove the responsible porcine epitopes include decellularization [11, 20, 57] and/or enzymatic cleavage [50, 51]. Promising outcomes were reported for small‐scale clinical studies of ACL reconstruction with enzymatically‐treated porcine bone‐patellar‐tendon‐bone (BPTB) however this xenograft is no longer clinically available [20, 51]. One limitation was that −70°C storage is required. Recently, a decellularization process has been developed that permits room temperature storage of porcine soft tissue grafts [11, 20, 57]. This process can be applied to porcine digital extensor tendon xenograft which has the required size and mechanical properties for use as a hamstring (HS) graft alternative for ACL reconstruction [40].

This human clinical study was designed to evaluate the performance and safety of a decellularized porcine digital extensor tendon xenograft (d‐pDET) over a 5‐year period. Our hypothesis was the safety and performance outcomes of this study would support this xenograft being a viable graft option for ACL reconstruction.

## MATERIALS AND METHODS

### Study design

This was a prospective, non‐comparative multicenter clinical investigation. Screening (baseline) was performed within 30 days prior to surgery with patients providing written informed consent prior to surgery. One surgeon per each of 7 clinical sites contributed cases to the study. The following Ethics Committees approved this study: NRES Committee West Midlands ‐ Edgbaston, REC Reference 15/WM/0101; EC of Hospital Bellvitge, Approval number AC032/15; EC of The Jimenez Diaz Foundation, Reference number EC 42‐15/HRJC; Bioethics Committee of the Regional Medical Council at Wielkopolska Medical Chamber, EC Approval number 129/2015; CEIC Hospital Clinico San Carlos, 15/206‐P; and EC of University Hospital of La Ribera issued ‐1 JUL 2015.

Initially designed as a 24‐month follow‐up study, it was later amended to include up to 60‐month follow‐up requiring a second written informed consent by the patient. Inclusion criteria included (1) men and women 18 years or older, (2) partial or complete tear of ACL requiring surgery, (3) passive flexion 120° and passive extension the same on both knees, (4) medial collateral ligament (MCL) injury Grade 2 or less, (5) osteoarthritis Grade 2 or less on the Kellgren Lawrence scale, and (6) ability to communicate meaningfully, and willingness and ability to comply with study procedures. Exclusion criteria included (1) Body Mass Index (BMI) > 35 kg/m^2^, (2) pregnant at screening and/or intending to become pregnant in the next 12 months, (3) abnormal degenerative osteoarthritis of the joint (e.g., International Cartilage Repair Society Grade III or higher) as determined by the baseline MRI scan, (4) previous ACL reconstruction on target knee, (5) current ACL injury to contralateral knee, and (6) meniscectomy consisting of removal of more than one‐third of the meniscus on the target knee.

### Xenograft description

**Figure 1:**
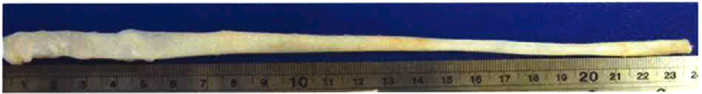
OrthoPure® XT decellularized porcine digital extensor tendon (d‐pDET) xenograft. (Reproduced with permission by Tissue Regenix Group, Leeds, UK.).

OrthoPure® XT (Tissue Regenix Group, Leeds, UK) is a decellularized xenograft derived from porcine digital extensor tendon (pDET) of the hind limb—Figure 1. The graft is decellularized with the proprietary dCELL® Technology (Tissue Regenix Group, Leeds, UK) that includes treatment at freezing temperatures, exposure to salt and nuclease solutions, and processing with a mild detergent to remove all viable cells and native components which have the potential to elicit an immune response [10, 11, 20, 57]. This produces a biocompatible and biomechanically appropriate acellular scaffold for cellular repopulation and eventual regeneration. The graft is suspended in normal saline (0.9%) in a blister package followed by sterilisation via 25 kGy gamma irradiation allowing room temperature storage. Size 8 grafts (8–9 mm diameter) were utilised consisting of two individual d‐pDET xenografts to create a 4‐strand, double‐looped, 8–9 mm diameter construct. This configuration has a failure load of 3,559 ± 394 N and a stiffness of 495 ± 47 N/mm (unpublished data) which compares to 416.4–4590.0 N and 192.9–861.0 N/mm for four‐strand cadaveric hamstring tissue, respectively [31]. The OrthoPure® XT xenograft has received regulatory approval via the CE Mark process for knee ligament, including ACL, reconstruction.

### Surgical procedure

The d‐pDET graft has the form and function of human hamstring graft. As such, the surgeons at each clinical site utilised their preferred surgical technique for using soft tissue grafts for ACL replacement using femoral and tibial tunnels with the major steps as follows. Meniscal defects, if present, were treated as necessary during the index procedure immediately prior to ACL reconstruction. The diameter of the d‐pDET was measured (e.g., graft sizing block or sizing tubes) to determine appropriate tunnel diameters to ensure optimal fit. Femoral and tibial tunnels were then prepared. The xenograft was passed through the tunnels and secured with an appropriate soft tissue fixation system (e.g., interference screws and suspensory devices). A final assessment of tension, stability and hardware position was performed prior to closing the surgical site.

### Rehabilitation

Rehabilitations guidelines were as follows: Days 1–10—A progressively increasing regimen including continuous passive movement, strengthening and range of motion exercises, and gradual weight bearing to achieve full extension, 120° flexion, and mobility with appropriate walking aids. Day 10–Week 6—Continued strengthening and stretching exercises adding early plyometrics, proprioceptive exercises, and hydrotherapy/swimming with the goal of straight leg raise with no lag and full range of motion. Weeks 6–12—Introducing rowing, road cycling, power walking, and jogging progressing as appropriate as well as core stability and strengthening exercises to reach a normal gait pattern with no anterior knee pain. 3–6 Months—Progression of mobility and function, plyometrics, jogging to running, and non‐contact sports to prepare for safe full return to sports or activities after 6 months.

### Clinical assessment

Clinical assessment was performed at screening and at 3, 6, 12 and 24 months then annually to 5 years. Evaluations performed included (1) arthrometric measurement of anterior tibial translation of both the target and contralateral knee taken with the GNRB® Knee Arthrometer (Genourob, Laval, France) [48] with a side‐to‐side (SSD) difference ≤ 3 mm considered clinically acceptable, > 3–5 mm denoting residual laxity, and >5 mm indicating an unfavourable outcome [18]. (2) Lachman test [26], (3) Pivot Shift test [26], (4) International Knee Documentation Committee (IKDC) subjective questionnaire [19], (5) Lysholm score [52], (6) Knee Osteoarthritis and Injury Outcome Score (KOOS) questionnaire including five subscales [44], and (8) intraoperative evaluation by the investigator of use of the xenograft including amount of material provided, ease of fixation, ease of implantation, and overall satisfaction, rated as excellent, good, fair, or poor.

Reconstruction failures were defined as cases meeting all three criteria: (1) SSD > 5 mm, (2) laxity as defined by the surgeon (Lachman or Pivot Shift tests), and (3) the patient experiencing a trend of functional instability demonstrated in more than one questionnaire.

All intraoperative and post‐operative adverse events were recorded. Our primary interest was to compare 2‐year and 5‐year outcomes with baseline so 3‐year and 4‐year outcomes are not reported here.

### Imaging assessment

MRI imaging was performed at 1.5 T field strength with (1) coronal T1 weighted sequence, (2) coronal proton density sequence with fat saturation, (3) sagittal T2‐weighted sequence, (4) sagittal proton density sequence, and (5) axial T2‐weighted sequence taken at screening, and 3, 6, 12 and 24 months. Assessment included evaluation of femoral and tibial tunnel widening with tunnel diameters measured 10 mm from the tunnel margin at the joint space, graft integrity, and ligamentization.

Graft integrity was graded as [38]:

1. No signal (no evidence of increased signal intensity) indicative of graft continuity and maintenance of collagen fibrillar structure.
2. Increased signal but fibres intact (presence of an increased T2 signal, but graft fibres are still intact).
3. Graft disruption (Graft is disrupted regardless of T2 signal intensity)
  1. Partial disruption
  2. Complete/full disruption
  3. Unable to determine

Ligamentization was graded as:

1. Absent: No evidence of complete ligamentization of the graft material.
2. Low signal on T1 and T2 weighted images consistent with the normal visual appearance of healthy, unreconstructed ACL.

### Statistical analysis

Comparisons at baseline and all follow‐up intervals were performed on a group basis utilising one‐way analysis of variance (ANOVA) followed by a post hoc Tukey test for continuous data and a Chi square test with a Bonferroni multiple comparisons adjustment for categorical data. Continuous data was compared for two groups with a two‐tailed Student t‐test. Significance was considered for *p* < 0.05.

## RESULTS

Forty‐three subjects were screened for study participation, of which two failed baseline eligibility and one withdrew consent prior to treatment. As such, 40 study patients underwent ACL reconstruction at seven investigational sites from December 2015 to September 2016. Mean age was 31.9 years (range: 18–48), with 70% (28/40) male, right side treated in 47.5% (19/40), and mean BMI was 24.7 kg/m^2^ (range: 17.9–34.2 kg/m^2^)—Table 1. Suspensory and interference screw fixation techniques were mainly utilised to stabilise the xenograft on the femoral and tibial sides, respectively, with bioabsorbable interferences screws used in 67.5% (27/40) of the patients.

**Table 1:** Patient demographics *n* = 40.

Parameters		Complete cohort
Gender	Male	28 (70%)
	Female	12 (30%)
Side	Right	19 (47.5%)
	Left	21 (52.5%)
Age (y) mean ± SD (range)		31.9 ± 8.7 (18–48)
BMI (kg/m^2^) mean ± SD (range)		24.7 ± 3.0 (17.9–34.2)
Follow‐up (m), mean ± SD (range)		41.9 ± 19.1 (6–60)

Abbreviations: BMI, body mass index; SD, standard deviation.

Preoperative MRI performed at screening identified 24 of 40 (60%) patients with meniscal tears, including 15 with vertical tears, 1 with a horizontal tear and a cyst, 4 with both vertical and horizontal tears, 1 with an oblique tear, 1 with an oblique and a vertical tear, 1 with a bucket handle tear, and 1 with a tear whose type was not described. Many of the tears identified by MRI were small and not clinically relevant. Hence, 11 (27.5%) patients underwent one‐third meniscectomy, including 6 medial (with the remaining tissue being normal in 3 and having degenerative changes in 3), 4 lateral (with the remaining tissue being normal in 3 and having degenerative changes in 1), and 1 both medial and lateral (with the remaining tissue being normal medially with a small tear laterally). No meniscal tears were treated with meniscal repair devices.

The investigators rated the amount of material, ease of fixation, ease of implantation, and their overall satisfaction as predominantly excellent (≥77% of cases) with the remainder good (≥23% of cases) for all subcategories.

**Figure 2:**
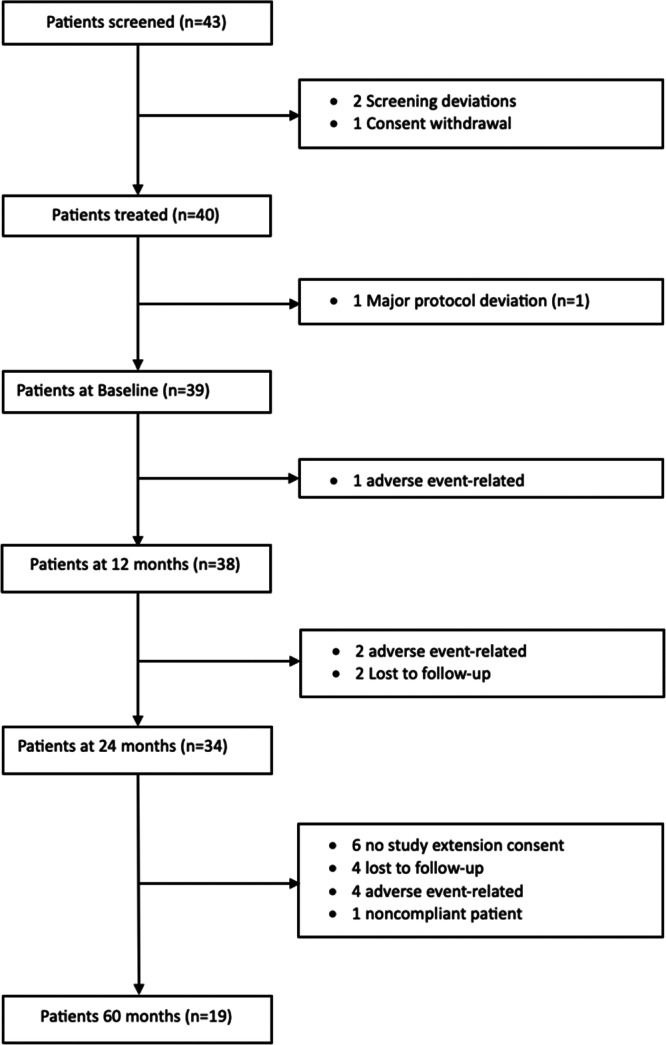
Flow chart showing the disposition of patients over the 60‐month term of the clinical study.

Figure 2 tracks subject disposition over time. One subject was treated with a single‐looped double‐stranded xenograft instead of a double‐looped, four‐stranded xenograft due to the size constraint of the intercondylar space. As this was a major protocol deviation, this subject was excluded from the analysis except for safety consideration. Hence, 39 subjects comprised the baseline population as regards performance (40 patients evaluated for adverse events) with 38, 34 and 19 remaining at 12, 24 and 60 months, respectively. Patient attrition was ascribed to lost to follow‐up (*n* = 6), revisions (*n* = 7), consent refusal for study extension to 5 years (*n* = 6) and noncompliance (*n* = 1).

### Clinical assessment

#### Arthrometer stability

**Figure 3:**
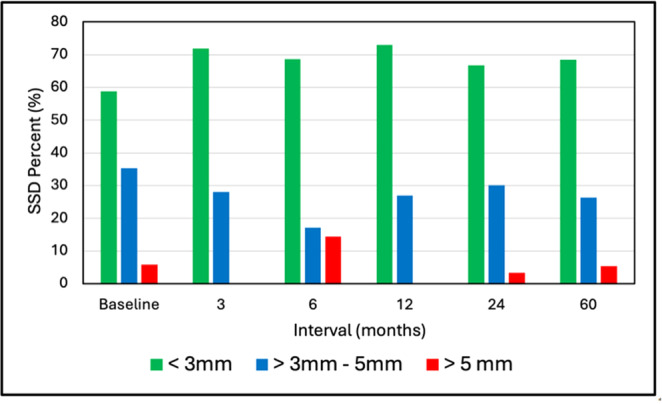
Side‐to‐side difference (SSD) stratified by magnitude range (%). *p* = 0.228 for distribution versus interval.

The mean SSDs were 2.72 ± 1.80 mm, 2.25 ± 1.55 mm, 2.12 ± 2.29 mm, 2.28 ± 1.59 mm, 2.49 ± 1.46 mm, and 2.07 ± 1.95 mm for baseline, 3, 6, 12, 24, and 60 months, respectively (*p* = 0.727). Figure 3 shows the SSD distribution stratified by magnitude, that is, ≤ 3 mm, > 3–5 mm, and >5 mm. The majority of patients had SSDs magnitudes of ≤ 3 mm followed by > 3–5 mm and >5 mm with no significant differences in distribution over time (*p*. 0.228).

#### Lachman test

**Figure 4:**
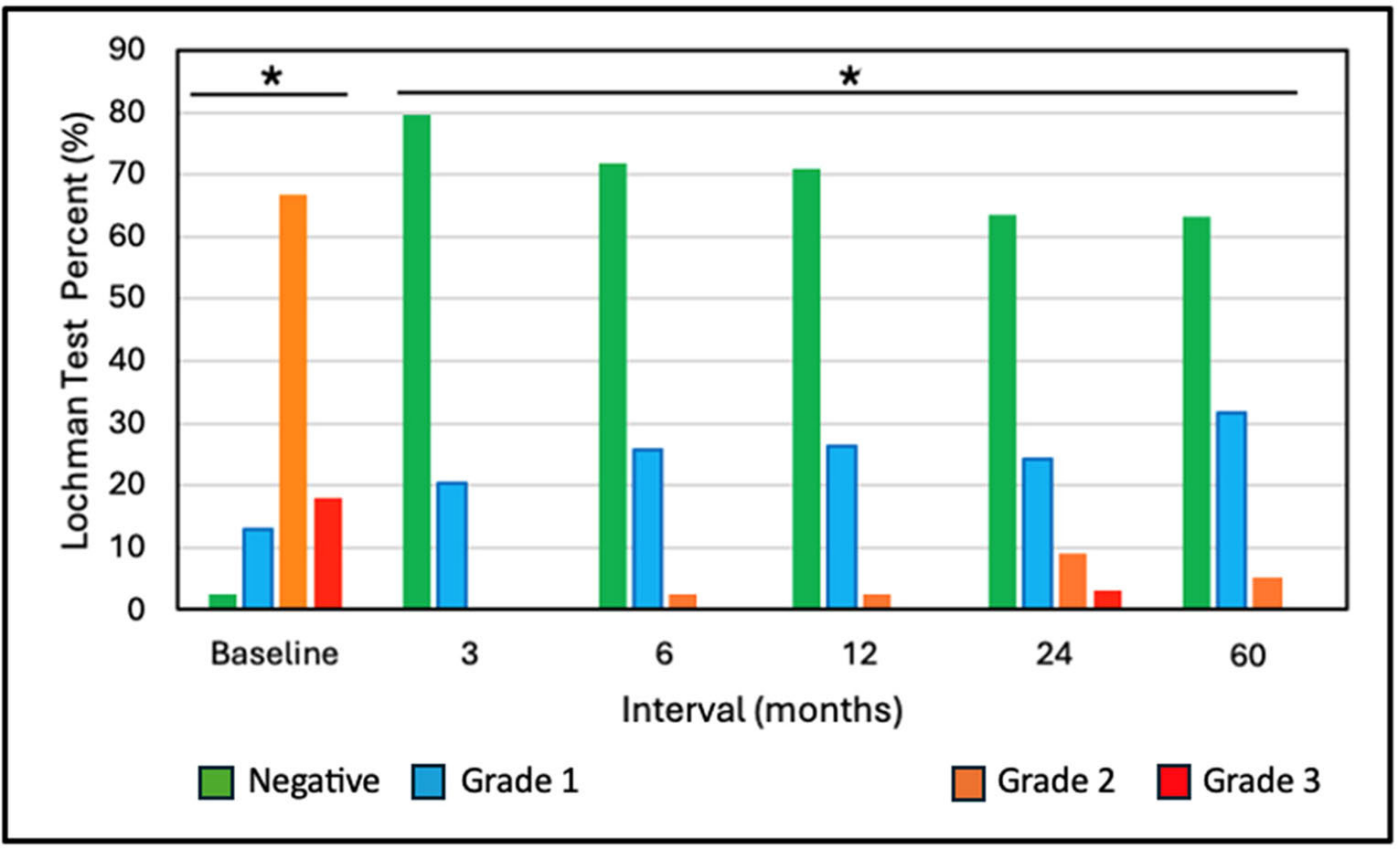
Lachman test outcomes (mean ± SD). *Groups significantly different than Baseline (*p* < 0.001).

Negative and positive grades 1, 2, and 3 Lachman test results at baseline were 3.0%, 12.1%, 63.6% and 21.2%, improving to 66.7%. 27.8%, 5.6% and 0.0% at 60 months, respectively—Figure 4. All follow‐up interval outcomes were significantly improved from baseline (*p* < 0.001).

#### Pivot shift test

**Figure 5:**
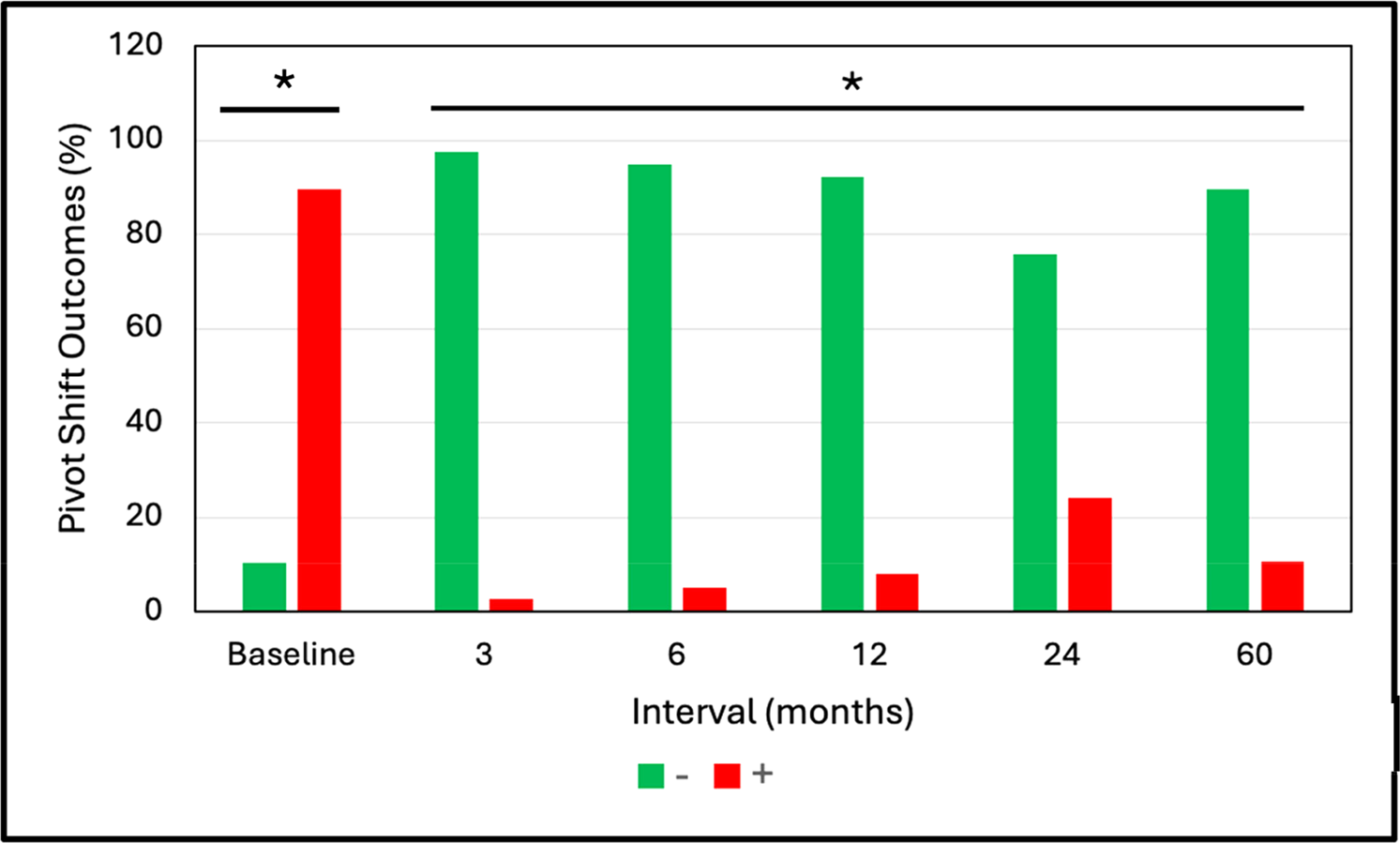
Pivot Shift test outcomes (mean ± SD). *Groups significantly different than baseline (*p* < 0.001).

Most patients at baseline had a positive pivot shift test (89.7%) while most at 3–60 months were negative (75.8%–97.4%)—with 60‐month outcomes being 89.5% of patients negative and 10.5% positive—Figure 5. Outcomes at all follow‐up intervals were significantly improved from baseline (*p* < 0.001).

#### IKDC subjective score

**Figure 6:**
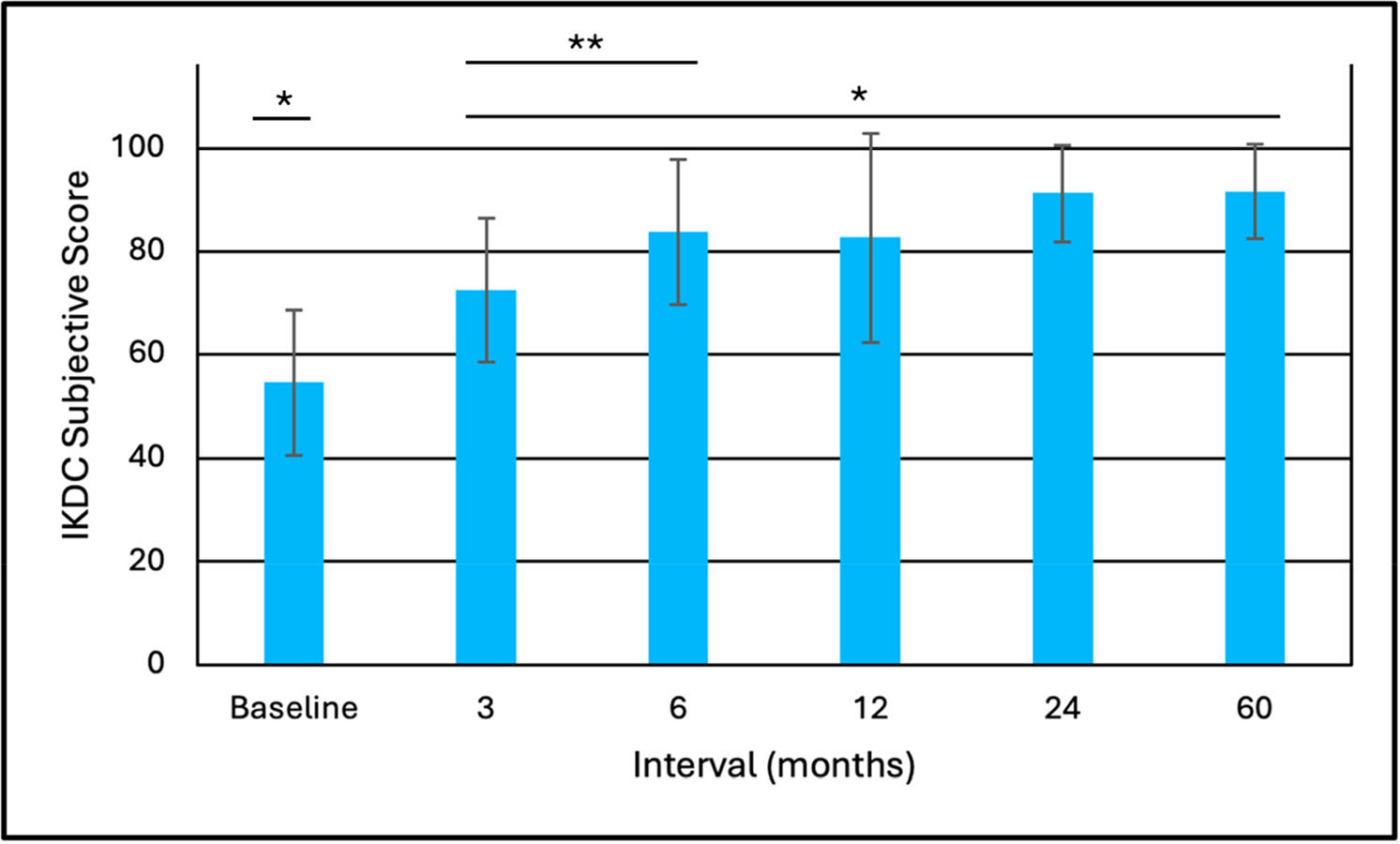
IKDC Subjective score outcomes (mean ± SD). *Comparison to Baseline (*p* < 0.001) **3 months vs. 6 months (*p* = 0.010). IKDC, International Knee Documentation Committee; SD, standard deviation.

The mean IKDC subjective scores at all follow‐up intervals were significantly greater than the baseline score (*p* < 0.001) with no significant differences from 6 to 60 months although there was a trend toward slight improvement reaching 91.6 ± 9.2 at 60 months—Figure 6.

#### Lysholm score

**Figure 7:**
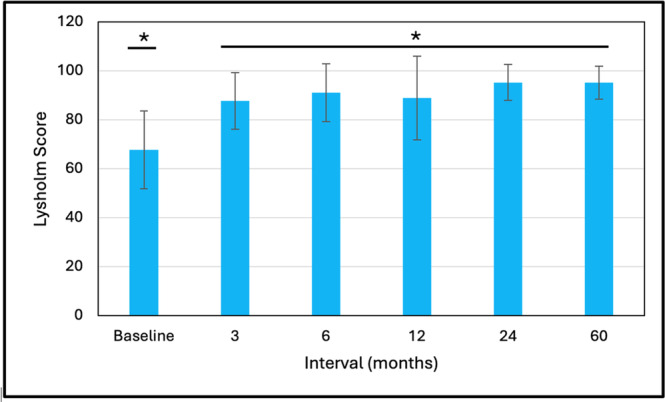
Lysholm score outcomes (mean ± SD). *Comparison to baseline (*p* < 0.001).

The Lysholm scores are summarised in Figure 7. The mean scores at all follow‐up intervals were significantly greater than the baseline score (*p* < 0.001) with no significant differences from 3 to 60 months and a final score of 95.2 ± 6.8.

#### KOOS score

**Figure 8:**
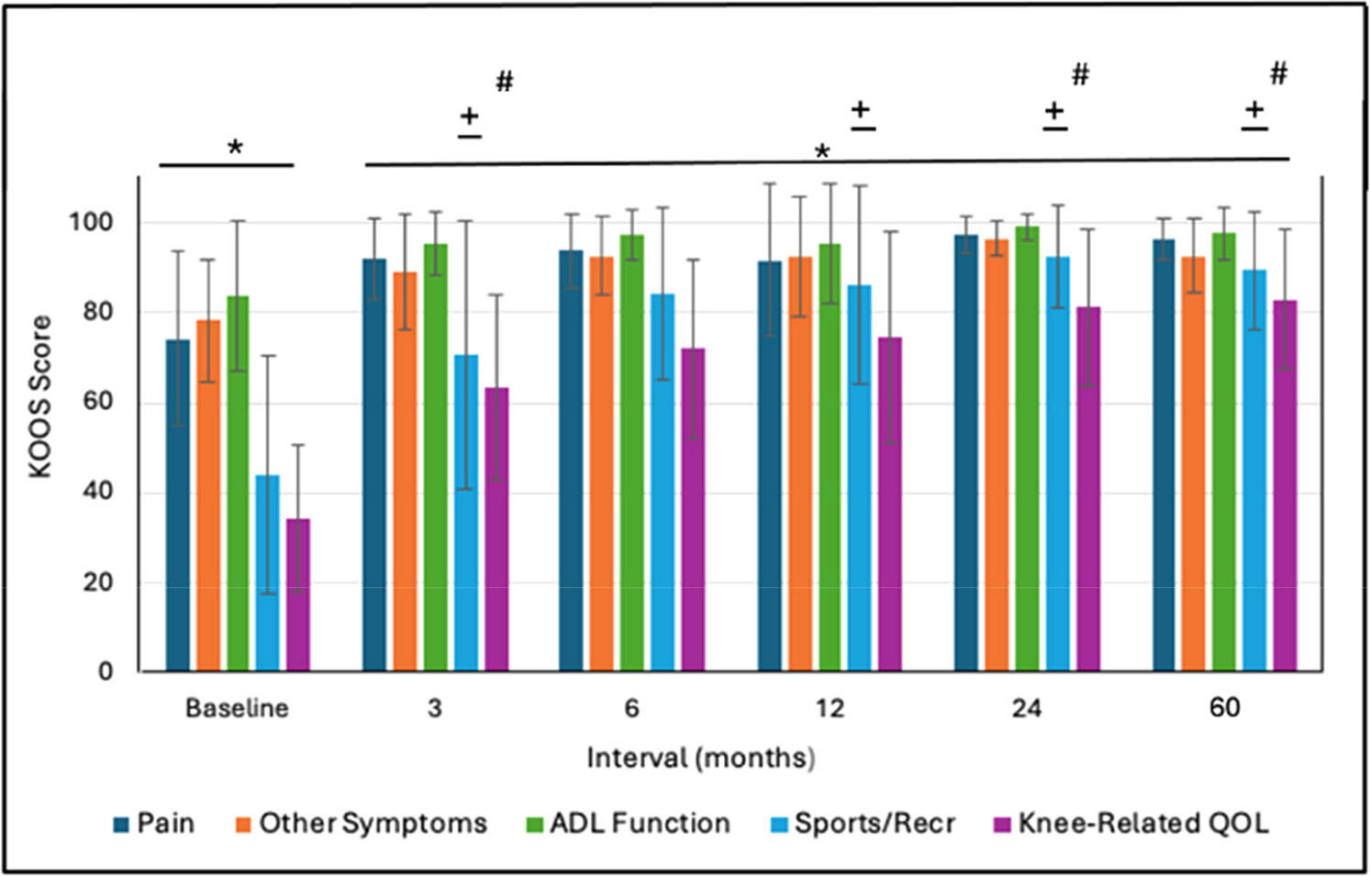
KOOS Score outcomes (mean ± SD). *Post‐op groups compared to Baseline (*p* < 0.001). ± Sport/recreation function at 12–60 months compared to 3 months (*p* < 0.032). # Knee‐related QOL at 24–60 months compared to 3 months (*p* < 0.005). KOOS, Knee Osteoarthritis and Injury Outcome Score; QOL, quality of life; SD, standard deviation.

All domains of the KOOS score (pain, other symptoms, ADL function, sports/recreation function, and knee‐related QoL increased significantly from baseline to 3 months and all subsequent follow‐up intervals (*p* < 0.001), reaching 96.7 ± 4.6, 92.8 ± 8.3, 97.7 ± 6.0, 89.5 ± 13.0 and 83.1 ± 15.5, respectively at 5 years—Figure 8. In general, there were no significant differences among the 3–60 month scores for the respective domains with the following exceptions: (1) the sport/recreation scores at 12, 24, and 60 months were significantly greater than the 3‐month score (*p* ≤ 0.032) and (2) the knee‐related QoL scores at 24 months and 60 months were significantly greater than the 3‐month score (*p* ≤ 0.0052).

### Imaging assessment

#### Bone tunnel widening

Among the 39 patients, the mean baseline femoral and tibial tunnel diameters as prepared were similar at 8.3 ± 0.6 mm and 8.6 ± 0.5 mm, respectively. Tunnel diameters at 24 months were available for 34 patients, showing the following mean diameter increases relative to baseline on a paired basis—femoral AP 31.3%, femoral ML 32.5%, tibial AP 32.6%, and tibial ML 36.0%.

#### Graft integrity

Graft integrity, as measured by MRI imaging, is listed in Table 2. From 3 to 24 months, the findings of “no signal” and “increased signal but fibres intact”, were in the range of 57.6%–76.9%. reflecting that most xenografts possessed structural integrity. The categories of “partial disruption” and “complete disruption” were 20.5%–30.3% and 2.6%–12.1%, respectively. Among the 3–24‐month intervals, there was no significant difference in the distribution of the graft integrity categories (*p* = 0.193).

**Table 2:** Graft integrity per MRI.

Graft integrity	No. Knees (%)			
	3 months	6 months	12 months	24 months
No signal	6 (15.4)	2 (5.1)	1 (2.7)	2 (6.1)
Increased signal but fibres intact	22 (56.4)	28 (71.8)	25 (67.6)	17 (51.5)
Partial disruption	8 (20.5)	7 (17.9)	8 (21.6)	10 (30.3)
Complete disruption	1 (2.6)	2 (5.1)	3 (8.1)	4 (12.1)
Unable to assess	2 (5.1)	0 (0.0)	0 (0.0)	0 (0.0)

*Note*: *p* = 0.193 for follow‐up interval comparisons.

**Figure 9:**
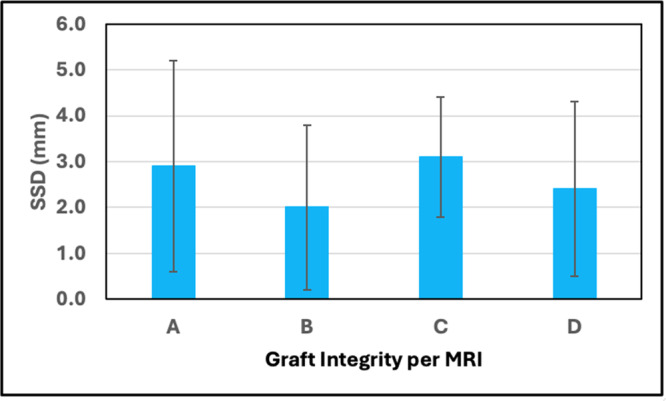
Side‐to‐side difference (SSD) vs. graft integrity status per magnetic resonance imaging (MRI). A = No signal, B = Increased signal but fibres intact, C = Partial disruption, D = Complete/full disruption.

Since patients had both MRI and SSD measured at the same postoperative intervals, the mean SSD corresponding to each graft integrity status was calculated across all patients and intervals. The following results were obtained – (1) no signal: 2.9 ± 2.3 mm, (2) increased signal but fibres intact: 2.0 ± 1.8 mm), (3) partial disruption: 3.1 ± 1.3 mm, and (4) complete/full disruption: 2.4 ± 1.9 mm. No trend was apparent ‐ Figure 9.

#### Ligamentization

Ligamentization via MRI was apparent in 2 of 38 patients (5.2%) at 12 months and 1 of 33 patients (3.0%) at 24 months. One of these two patients sustained a re‐rupture at 12 months and exited the study with other patient retaining its ligamentization status at 24 months.

### Adverse events

Adverse events were evaluated based upon 40 patients which included the patient with the major protocol deviation.

No patients without prior retear met the criteria for reconstruction failure so were only considered to have had a primary retear adverse event. Six retears (15.0%) occurred including 2 traumatic, 3 atraumatic, and 1 unknown, occurring at a mean 15.3 months (range: 5.5–43 months) after surgery. The ages of the retear (*n* = 6) and intact (*n* = 34) groups were 25.5 ± 5.4 years and 33.0 ± 8.7 years (*p* = 0.049), the BMIs were 23.7 ± 3.9 kg/m^2^, and 24.9 ± 2.9 kg/m^2^ (*p* = 0.489), and genders were 100% male and 64.7% male (*p* = 0.153), respectively. Among the two cases of traumatic retear, the preceding MRI‐based graft integrities were complete disruption and increased signal but fibres intact, respectively. The corresponding graft integrities for the 3 atraumatic retears were increased signal but fibres intact, partial disruption and complete disruption, respectively. Finally, the retear with unknown cause was associated with an integrity of increased signal but fibres intact. Seven revisions (17.5%, 7/40) were performed, including the six patients that experienced graft rupture (one patient withdrew consent and underwent revision outside of the study), and one revised for pain and instability at 15 months. Three of the 27 patients (11.1%) that received bioabsorbable screws reacted adversely to the material, developing mild effusion and granuloma (*n* = 1), or mild synovitis (*n* = 2) which resolved after debridement and removal of residual screw removal at 12–18 months. There were 10 additional patients with effusions (25%), eight of which exhibited mild effusions at 0.2–1.8 months and two exhibiting effusions (1 mild and 1 moderate) at 3.2–4.5 months. One (2.5%) infection/osteomyelitis occurred at the tibial tunnel that was treated surgically at 14 months. Intermittent arthralgia was reported for 1 (2.5%) patient at 49 months. A fragment of metal guide used to deliver a bioabsorbable interference screw was retained in one patient (2.5%) and was surgically removed at 3 months.

## DISCUSSION

The limitations of autograft, allograft, and synthetic grafts for ACL reconstruction have made the development of a viable alternative desirable. We investigated the clinical performance and safety of a novel ACL xenograft comprised of decellularized porcine digital extensor tendon that has similar form and mechanical properties to human 4‐strand hamstring graft and can be stored at room temperature. The surgeons rated their intraoperative experience with the xenograft and overall satisfaction as excellent or good. Prior experience with hamstring grafts likely helped minimise the learning curve. This is important because, on average, each surgeon only performed approximately six procedures.

We found significant improvements (*p* < 0.001) from baseline to 3–6 months in various outcome measures that were maintained, or further improved, to 60 months. This trend of early recovery followed by maintenance is similar to the recovery patterns commonly reported following ACL reconstruction [3]. Comparison of our 5‐year mean outcomes with those reported at follow‐up from a systematic review by Agarwalla et al. [3] utilising a variety of common graft types are, respectively: Subjective IKDC 91.6 versus 86, Lysholm 95.2 versus 91, KOOS Pain 96.7 versus 90, KOOS Symptoms 92.8 versus 85, KOOS ADL 97.7 versus 96, KOOS Sports 89.5 versus 81, and KOOS Quality of Life (QoL) 83.1 versus 71. Our 5‐year Lachman test outcomes were 66.7% negative (Grade 0), 27.8% Grade 1 positive, and 5.6% Grade 2 positive and pivot shift outcomes of 88.9% negative and 11.1% positive (not stratified by grade). The meta‐analysis of Mouarbes et al. [32] found Lachman outcomes at follow‐up with quadriceps autograft of 81.2% negative and 18.8% positive and pivot shift values of 84.8% negative and 15.2% positive. Krishna et al. [24] performed a 2‐year prospective, randomised controlled study comparing 4‐strand versus 5‐strand hamstring autografts. Final Lachman test outcomes were 50.0% and 46.4% negative with the remainder mainly positive grade 1, respectively. Their corresponding values for the pivot shift test were 46.4% and 53.6% negative with remainder mostly Grade 1 positive. Thus, overall, the performance outcomes at follow‐up for d‐pDET are comparable to those published for a variety of traditional graft configurations.

The mean arthrometric SSD's between the treated and the normal knees at baseline and all follow‐up intervals were < 3 mm with no significant differences over time. The mean SSD at 5 years was 2.07 ± 1.95 mm categorised as 66.7% ≤ 3 mm, 27.7% 3–5 mm, and 5.6% > 5 mm. A meta‐analysis by Freedman et al. [13] following use of patellar tendon and hamstring autografts yielded 79.0% and 73.8% < 3 mm, 15.4% and 19.4% 3–5 mm, and 5.6% and 6.8% > 5 mm, respectively. Mouarbes et al. [32] performed a meta‐analysis of quadriceps tendon (QT) autograft usage and found a mean SSD of 1.72 mm with 73.8% of knees ≤ 3 mm and 26.2% > 3 mm. As such, the SSD of knees following ACL reconstruction with d‐pDET was comparable to published values for various autografts. It is reasonable to assume that laxity, as represented by SSD, would increase with diminished graft integrity. However, the mean SSDs for the four integrity categories that spanned from no signal to complete disruption were all in the range of 2.0–3.1 mm, with no apparent trend. This shows that, in our study, there was little correlation between MRI‐based graft integrity and laxity.

Bone tunnel widening following ACL reconstructive surgery is well documented and has been reported to occur in 25%–100% of femoral tunnels and 29%–100% of tibial tunnels [2, 49, 58]. Femoral tunnels have been known to enlarge 3%–45% and tibial tunnels 11%–45% [2]. As regards 4‐strand gracilis/semitendinosis autografts, diameter increases of 29.2%–40% and 23.2%–30.3% have been reported in the femoral and tibial tunnels, respectively [7, 25]. The mechanism is unclear but is likely multifactorial, including several mechanical and biological aspects which may help explain the wide variance reported in the literature [58]. In general, the rate of widening is greatest during the first 6 weeks after surgery with minimal change between 3 months and 2 years and a decrease seen at 3 years [58]. At least in the short term, there appears to be no effect on laxity or increased failure rates with one of the main concerns being an increase in complexity of revision surgery due to bone loss near the joint [2, 58]. In the present study, nearly 90% of the patients experienced widening of both tunnels with the average 24‐month increases being in the range of 31%–36%. Consequently, our study shows no new concerns arising from use of the d‐pDET xenograft.

An immunological reaction can ensue when a discordant xenograft is placed in a host, triggering the complement cascade and leading to xenograft rejection and failure [14, 50]. The potential for porcine xenograft to elicit an immunological response in humans can be mitigated by extracting the carbohydrate α‐Gal epitope associated with glycolipids and/or glycoproteins of various nucleated cell membranes as well as removing remnant DNA which has been implicated in inflammatory reactions [4, 14, 15]. The combined features of decellularization and enzymatic removal of remnant DNA is the basis of the dCell® processing of the xenografts used in our study.

ACL xenograft rejection manifests clinically as a chronic inflammatory reaction reflected by recurrent/ongoing knee effusion and/or synovitis [21, 35, 50, 53]. In our study, 11 of 40 patients (27.5%) developed effusions and 2 of 40 (5%) developed synovitis. Three patients exhibited adverse reactions to bioabsorbable screws, a known complication to such materials, including one case of effusion and two of synovitis [36]. Consequently, there were 10 (25.0%) occurrences of effusion (9 mild and 1 moderate) unrelated to bioabsorbable screws. Eight of these patients exhibited mild effusions at 0.2–1.8 months and two exhibited effusions (1 mild and 1 moderate) at 3.2–4.5 months. Joint effusions are not uncommon during the first 3 months following reconstructive ACL surgery, with Kikuchi et al. [22] reporting a rate of 9.1% 3 months after ACL reconstruction with autologous hamstring graft. This suggests that our early effusions, most of which were mild, may not reflect immunogenic response. Kikuchi et al. [22] found that the presence of effusion at 3 months was a risk factor for future ACL injury. Among the patients in our study without effusion and with effusion, re‐rupture rates were not significantly different at 13.8% (4/29) and 18.2% (2/11), respectively (*p* = 0.729). Consequently, there is no overt evidence of a clinically relevant immunogenic response.

Graft retear has a multifactorial aetiology with many risk factors including male gender, younger age, high activity level, trauma, graft and fixation type, and others. Short‐ to mid‐term retear rates of 1.8%–10.4% are not uncommon and can reach 40% [29, 42, 43, 56]. In the present study, retear occurred in 6 of 40 (15.0%) patients with at least two being associated with trauma. There was no significant difference in the proportion of male patients (100% vs. 64.7%, *p* = 0.153) or BMI (23.7 ± 3.9 kg/m^2^ vs. 24.9 ± 2.9 kg/m^2^, *p* = 0.489) and a minimally significant difference in patient age (25.5 ± 5.4 years and 33.0 ± 8.7 years, *p* = 0.049 in the retear and intact groups, respectively. Thus, the overall retear rate as well as the tendency to occur in young males is supported by the literature. Interestingly, there was no correlation between the preceding MRI‐based determination of graft integrity with subsequent retears.

Ultrastructural, morphological and biomechanical differences exist between tendons and ligaments [6, 41]. Animal and human studies have shown that, over time, the transplanted tendonous ACL graft undergoes ligamentization by acquiring the characteristics of the native ACL [6, 16, 47]. This process is a continuum that includes neovascularization, infiltration of host fibroblasts, and the formation of an organised collagenous extracellular matrix. Macrophages infiltrate the graft and gradually remove the graft material as it is replaced with host tissue, resulting in an initial reduction in mechanical properties that later rebound [23, 40]. This process occurs more slowly in humans than in animals and can require years to complete, although some differences may persist [6]. Autograft bone patellar tendon bone and hamstring studies in patients have shown histological and arthroscopic evidence ranging from an immature state of remodelling at 12 months [1] to a state of close approximation to the normal ACL occurring from 1 to 3 years [12, 39, 45]. A systematic review of the assessment of graft maturity by van Groningen et al. [54] showed that biopsy and histological evidence support an ongoing process of remodelling at 12 months, with MRI evidence being equivocal due to the heterogeneity of MRI methodology and technical restrictions. A sheep study compared d‐pDET with sheep hamstring autograft for ACL replacement utilising bioabsorbable screw fixation at 3 and 6 months [40]. Both grafts showed a reduction in initial mechanical properties at 3 months which then increased and were statistically indistinguishable at 6 months as well as displayed multifocal calcification, ossification, Sharpey's fibre formation, and screw osseointegration at both intervals. In our study, biopsy and histological evaluation of the xenograft over time was not performed because it was not included in the study protocol, so MRI was used as a less effective alternative to examine its structure [9]. Low signal on T1 and T2 weighted images consistent with the normal visual appearance of healthy, unreconstructed ACL was used as evidence for ligamentization, showing ≤ 5% of the patients meeting this criterion at 12 and 24 months [34]. There was only a single occurrence of retear (traumatic) from 2 to 5 years with all 19 patients at 60 months having acceptable clinical outcomes, suggesting that the d‐pDET xenograft was sufficiently mature and stable after 2 years to provide a lasting femoral‐ACL‐tibial construct.

In summary, ACL reconstruction performed with d‐pDET xenograft yielded clinical results that were in substantial alignment with those obtained with traditional grafts. Outcomes reflective of stability, pain, and overall function significantly increased by 3–6 months, and were maintained to 60 months. The most significant complication was retear, however this was limited to young males which are known to be a high‐risk group, and its rate of occurrence was well within that described in the literature. Although MRI data provided only limited evidence of ligamentization by 24 months, the 60‐month data suggest that the xenograft had matured to provide lasting function. There were no apparent clinically relevant immunogenic responses to the xenograft. Overall, our results suggest an acceptable safety and performance profile for the d‐pDET xenograft.

As with any initial clinical study of an investigational device, patient recruitment can be difficult for a variety of reasons, including reluctance to undergo a procedure that has not yet been clinically proven. In the case of porcine‐derived xenograft religious and/or cultural barriers can exist as well. Nevertheless, as the body of clinical evidence continues to grow for using d‐pDET xenograft for ACL reconstruction, it is anticipated that this graft will become an important component in the surgeons' armamentarium.

Strengths of this study include being multicenter and permitting the surgeons to utilise their preferred surgical techniques reflective of real‐world surgical practice, follow‐up to 5 years, and having a wide array of metrics to characterise different aspects of clinical outcome. Limitations include a small study population at 60 months, exacerbated by refusal of six subjects to consent to study extension after 2 years, and no MRI data after 2 years. Future investigation should include a multicenter long‐term study with assessment of a larger number of patients.

## CONCLUSIONS

Decellularized porcine digital extensor tendon xenograft was developed to circumvent the limitations of autograft and allograft for ACL reconstruction. Its initial 5‐year clinical safety and performance profile suggests its suitability for ACL reconstruction.

## AUTHOR CONTRIBUTIONS

Neil Hunt provided substantial clinical interpretive insight, draft revision, and approval of final draft. Gabriel Oliver provided substantial clinical interpretive insight, draft revision, and approval of final draft. Alberto Francés Borrego provided substantial clinical interpretive insight, draft revision, and approval of final draft. William S. Pietrzak provided data analysis, initial manuscript draft, and approval of final draft.

## CONFLICT OF INTEREST STATEMENT

Neil Hunt paid consultant to TRX Orthopaedics Limited (Leeds, UK). Principal Investigator of OrthoPure® XT graft clinical study. Gabriel Oliver principal Investigator of OrthoPure® XT graft clinical study. Alberto Francés Borrego principal Investigator of OrthoPure® XT graft clinical study. William S. Pietrzak paid consultant to CellRIght Techniologies, LLC (Universal City, Texas, USA).

## ETHICS STATEMENT

Written informed consent was obtained from all patients before they were enroled to the clinical study. Permission to reproduce Figure 1 obtained from Tissue Regenix Group, Leeds, UK.

**Table:** 

Country	Name	Ethics Committee (EC)	Approval date
UK	Clifton Park Hospital, York	NRES Committee West Midlands ‐ Edgbaston, REC Reference: 15/WM/0101	23‐Apr‐15
Spain	Hospital Universitario de Bellvitge, Barcelona	EC of Hospital Bellvitge Approval number AC032/15	04‐Jun‐15
	Hospital Infanta Elena de Madrid, Valdemoro	EC of The Jimenez Diaz Foundation Ref: EC 42‐15/HRJC	12‐Nov‐15
	Hospital Universitario De La Ribera, Valencia	EC of Hospital Universitario De La Ribera	25‐Jun‐2015
	CEIC Hospital Clinico San Carlos	CEIC Hospital Clinico San Carlos, 15/206‐P	21‐May‐2015
Poland	Regional Medical Council at Wielkopolska Medical Chamber	Bioethics Committee of the Regional Medical Council at Wielkopolska Medical Chamber, EC Approval number 129/2015	09‐Sep‐2015

## Data Availability

The data that support the findings of this study are available from the corresponding author upon reasonable request.
